# Elimination of Active Trachoma after Two Topical Mass Treatments with Azithromycin 1.5% Eye Drops

**DOI:** 10.1371/journal.pntd.0000895

**Published:** 2010-11-23

**Authors:** Abdou Amza, Pablo Goldschmidt, Ellen Einterz, Pierre Huguet, Celine Olmiere, Philippe Bensaid, Lucienne Bella-Assumpta

**Affiliations:** 1 Faculté des Sciences de la Santé, Niamey, Niger; 2 Laboratoire du CHNO des Quinze Vingts, Paris, France; 3 Kolofata District Health Service, Kolofata, Cameroon; 4 Laboratoires THEA, Clermont-Ferrand, France; 5 Ophtalmo Sans Frontières, Luçon, France; 6 Ministry of Health, Yaounde, Cameroon; University of California San Francisco, United States of America

## Abstract

**Background:**

Following an epidemiological study carried out in 2006 showing a high prevalence of blinding trachoma in the Far North Region of Cameroon, a trachoma elimination programme using the SAFE strategy was initiated: three yearly trachoma mass treatments were to be performed.

**Methodology/Principal Findings:**

The entire district population (120,000 persons) was treated with azithromycin 1.5% eye drops in February 2008 and January 2009. To assess the effect of treatment on the prevalence of active trachoma, three epidemiological studies were conducted on a representative sample of children aged between 1 and 10 years. The first study was performed just prior to the first treatment, the second just prior to the 2nd treatment and the third one, one year later. The prevalence of active forms of trachoma (TF + TI) dropped from 31.5% (95%CI 26.4–37.5) before treatment to 6.3% (95%CI 4.1–9.6) one year after first treatment; a reduction of nearly 80%. One year after the second treatment, the prevalence decreased to 3.1% (95%CI 2.0–4.9), a total reduction of 90%. Furthermore, there were no more TI cases (only TF). There was no report of serious or systemic side effects. Tolerance was excellent.

**Conclusions/Significance:**

Active trachoma mass treatment with azithromycin 1.5% eye drops is feasible, well tolerated, and effective.

## Author Summary

Trachoma is the leading cause of infectious blindness worldwide, accounting for 1.3 million cases of blindness. Although it has disappeared in many regions of the world, trachoma is still endemic in Africa, Eastern Mediterranean, Latin America, Asia, and Australia. The WHO has currently set a target of 2020 for controlling trachoma to a low enough level that resulting blindness will not be a major public health concern. Topical tetracycline was for a long time the recommended treatment for active trachoma, but compliance to the regimen is extremely poor. Azithromycin has properties that make it an ideal treatment for *Chlamydia trachomatis*: high efficacy, intracellular accumulation, and a long tissue half-life. There is now a new mass treatment of trachoma by azithromycin 1.5% eye drops which is as effective as the oral route. In the test health district of Kolofata, Cameroon, the prevalence of trachoma among children dramatically decreased from 31% to less than 5% after 2 treatments. A third treatment was performed in January 2010. An epidemiological surveillance is implemented to see if this removal will be permanent. It also avoids misuse of oral azithromycin and the eye drops are directly treating the site of the infection.

## Introduction

Trachoma is caused by *Chlamydia trachomatis* and it is spread by direct contact with eye, nose, and throat secretions from affected individuals or by contact with objects, such as towels and/or washcloths, which have had similar contact with these secretions [1], [2]. Flies can also be a route of mechanical transmission [1]. Children are the most susceptible to infection but the blinding effects or more severe symptoms are often not felt until adulthood.

Infection is frequently passed from child to child but also from child to mother. Indeed, women are nearly two times [3] more affected than men by trachoma and trichiasis, probably because one of the primary activities of girls is taking care of their younger family members. This activity continues into adulthood, with women carrying the main responsibility of caring for children.

Trachoma remains the leading infectious cause of blindness in the world [4]. Mass oral azithromycin distribution has been used in several programs. At present, azithromycin is available in a 1.5% eye drop formulation in Europe, Maghreb and French speaking African countries. The National Blindness Prevention Programme and a Vision 2020 plan were established as a result of the prevention policy of the Republic of Cameroon., In December 2006, in the Kolofata Health District (Far North Cameroon), a study assessing the prevalence of active and scarring trachoma, signalled the presence of endemic trachoma with significant blinding potential [5]. The National Blindness Prevention Programme decided to plan an elimination program by implementing the SAFE (Surgery, Antibiotics, Facial cleanliness, and Environmental change) strategy [6], addressing the “A” (antibiotic) component by conducting mass treatment targeting the entire district population and using azithromycin 1.5% eye drops. The objective of this study was to assess the feasibility, tolerance and effectiveness of repeated topical mass treatment with azithtromycin 1.5% eye drops, used for the first time on a large scale to reduce the prevalence of active forms of trachoma in a population. The first year, mass treatment gave promising results with a decrease in trachoma prevalence of more than 25% (from 31.5% to 6.3%) [7]. This article presents results after two rounds of treatment.

## Methods

### Mass treatment campaign

**Figure 1:**
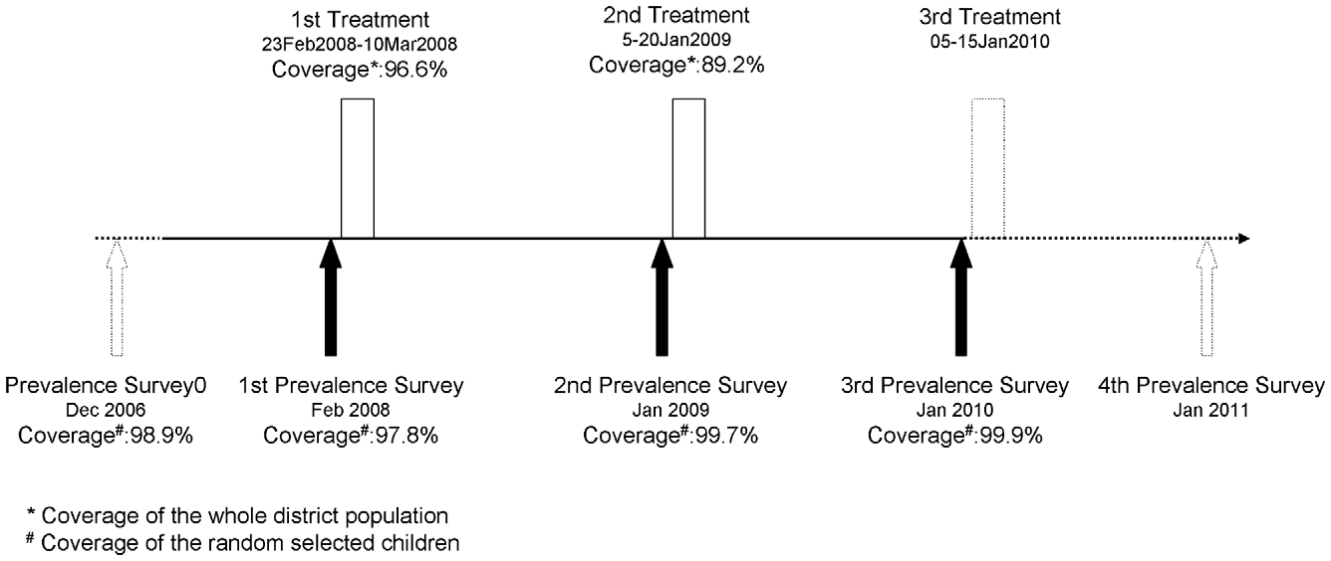
Flow chart of the study.

In accordance with the WHO recommendations [8], the trachoma control programme in the Kolofata Health District called for one mass treatment per year for three years. The treatment consisted in one instillation of azithromycin 1.5% in both eyes in the morning and in the evening during three consecutive days. The study received authorisation from the Cameroon Ministry of Public Health in February 2008. The first round of treatment began on 23 February 2008 and ended on 10 March 2008, and the second one was undertaken between the 5th and 20th of January 2009 (Figure 1).

Each year, Théa Laboratories donated 120,000 complete treatments (720,000 single doses) of azithromycin 1.5% eye drops, and sent them by air from Europe to Yaoundé, Cameroon, and by train and truck from Yaoundé to Kolofata.

The target population was all residents of the Kolofata Health District [9]. The entire population was treated, but only children between 1 and 10 year olds were examined for active trachoma. During the 15 days preceding the beginning of treatment, the local community health workers, helped by a literate second-level community health worker, conducted an exhaustive door-to-door census of all residents of the Kolofata Health District. Each of the 250 local community health workers was assigned a village or neighbourhood of 400 to 500 residents.

The local community health workers then administered treatment by visiting each household morning and evening for three consecutive days. Ophthalmic nurses were supervising each day the mass treatment in the different villages. A briefing was organized in Kolofata hospital each evening where the supervisors reported day after day their assessment of the mass treatment.

### The studies

As described by Huguet [7], among children aged between 1 and 9 year olds (up to their 10th birthday), three descriptive cross-sectional studies, to assess the effectiveness of treatment on the prevalence of active forms of trachoma in the population (trachomatous inflammation—follicular (TF) and/or trachomatous inflammation—intense (TI) [10], [11]), were conducted in the Kolofata Health District. The first was conducted prior to treatment in February 2008, the second prior the second treatment in January 2009, and the third in January 2010, one year after the second treatment (Figure 1). The standard WHO protocol for trachoma prevalence surveys was used [12].

The population studied was chosen at random and was based on the exhaustive list of villages and demographic statistics gathered in 2006 for the national census [9] and revaluate annually by a census at Kolofata sanitary district level only (118,617 inhabitants in 2009).

Assuming a prevalence of less than 5% at the end of the study (one year after the third treatment), it was necessary to include 2,400 children between 1 and 10 years in the study to obtain a precision of approximately 1.5% with a two-sided 95% CI and a cluster effect of 4 [13]. The 2,400 children were divided into 40 clusters, with 60 children per cluster chosen randomly.

All target-population residents (defined as children over 1 year and less than 10 years old [up to their 10th birthday] who had lived in the village for at least six months prior to the study date) in randomly selected households were registered and included in the population to be examined. When a family had left the community more than 6 months before the visit and the household remained empty, that household was replaced by the household nearest to it. A household that was only “temporarily” empty (less than 6 months) was not replaced. The research team returned up to three times to examine any subject absent during the preceding visit. If after the third visit the missing person was not found, that person was declared absent and not replaced.

Any family in the selected population whose head of household refused consent to participate in the study was not replaced [12]. This population corresponds to the enumerated population. The examined population corresponds to the population effectively examined.

A two-day training session helped assure standardization of procedures for conducting the census, examining subjects, and collecting and recording data. A post-training test was conducted on 50 trachoma patients to determine whether the trainee had mastered the WHO simple grading system and to confirm that each future examiner had a concordance of more than 80% with an expert examiner for each key sign. A pilot study was conducted in two villages not included in the current studies.

All children included in the study were examined by a senior nurse who everted the upper eyelid and examined the conjunctiva with a 2.5 magnifying glass and a torch held by an assistant who also recorded the data. The examiner changed gloves after the examination of each patient. Before examining the next person, the examiner verified that the assistant had filled out the study sheet in accordance with study protocol guidelines.

Data were compiled and analysed using EPIINFO 6 software. Estimated confidence intervals took into account the composition of sample clusters.

### Ethics Statement

All subjects provided informed consent. As people were illiterate, informed consent was read to people and if they agreed to participate the participant or legally acceptable representative put their fingerprints on the informed consent and a literate witness signed on behalf of the participant. National ethics committee of Yaounde approved the way to collect consent and the study before the beginning of the study.

## Results

### Results of the mass treatment campaign using azithromycin 1.5% eye drops

During the first annual treatment, azithromycin 1.5% eye drops were administered by the local community health workers to 111,340 of the 115,274 people counted in the census (coverage 96.6%) [7]. During the second round of treatment 105,802 people (45,288 adults and 60,514 children; 50,846 males and 54,956 females) received the full 6-dose treatment of azithromycin eye drops. In addition, 41,376 doses were administered to others who did not complete the full 6-dose treatment i.e. to people who were absent during at least one of the treatment administration visit.

The number of children examined during the study relative to the number counted in the random selected population is presented in Table 1.

**Table 1:** Survey participation: children >1 year and <10 years.

	1st prevalence survey	2nd prevalence survey	3rd prevalence survey
Examined children	2517	2404	2582
Random selected children	2570	2411	2585
% participation	97.9%	99.7%	99.9%

During the study, age and sex distributions were similar in the sample populations before and after treatments (p>0.05) (Table 2).

**Table 2:** Comparison of the distribution of children included in the survey in 2008, 2009 and 2010 by age and sex.

	1st prevalence survey		2nd prevalence survey		3rd prevalence survey	
	Number	%	Number	%	Number	%
1–4 years	1391	55.3	1332	55.4	1386	53.7
5–9 years	1126	44.7	1072	44.6	1196	46.3
Male	1280	50.9	1236	51.4	1316	51
Female	1237	49.1	1169	48.6	1266	49
Total	2517	100	2404	100	2582	100

### Prevalence of trachoma before and after treatment among children aged between 1 and 10 years

In February 2008, before all treatment, the prevalence of active forms was estimated to be 31.5% (95%CI 26.4–37.5). One year after the first mass treatment (January 2009), this prevalence dropped significantly to 6.3% (95%CI 4.5–8.6) (p<0.0001). One year after two rounds of topical treatment (January 2010), prevalence dropped to 3.1% (95%CI 2.0–4.9) (p<0.0001) (Table 3), a decrease of 90%.

**Table 3:** Prevalence of active trachoma (TF, TF/TI and TF + TF/TI) before and after treatment among examined children aged between 1 and 10 years.

	TF		TF/TI		TF + TF/TI	
	n	%	n	%	n	%
1st prevalence survey N = 2517	603	24%(95%CI: 20.7–27.5)	190	7.5%(95%CI: 5.7–10.0)	793	31.5%(95%CI: 26.4–37.5)
2nd prevalence surveyN = 2404	140	5.8%(95%CI: 4.1–8.0)	14	0.5%(95%CI: 0.13–1.6)	154	6.4%(95%CI: 4.5–8.6)
3rd prevalence surveyN = 2582	81	3.1%(95%CI: 2.0–4.9)	0	0%(95%CI: 0.0–0.8)	81	3.1%(95%CI: 2.0–4.9)

**Figure 2:**
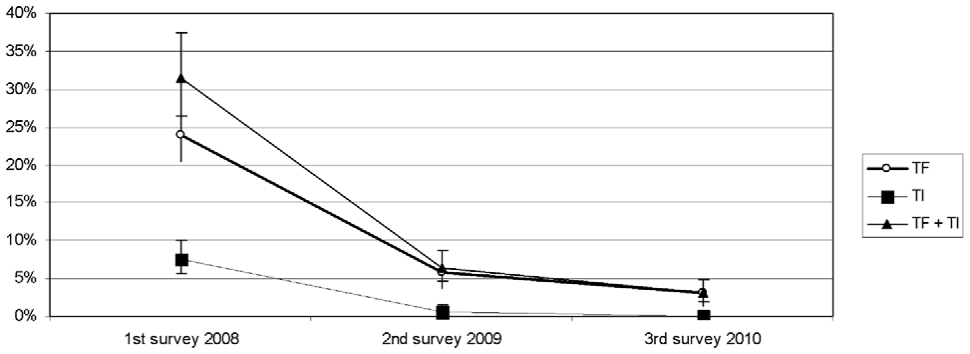
Prevalence of active trachoma in children aged between 1 and 10 years.

The prevalence of TF in the study sample was estimated to be 24% (95%CI 20.7–27.5) before treatment, it decreased significantly to 5.8% (95%CI 4.1–8) one year after first annual treatment (p<0.0001) and again significantly (p = 0.0001) to 3.1% one year after the second round of treatment (Table 3), either a decrease of 87%. The prevalence of TI was estimated to be 7.5% (95%CI 5.7–10) before treatment and disappeared after two annual treatments and (0.5% after 1^st^ treatment (p<0.0001) and 0% after second one (p = 0.0005) (Table 3) (Figure 2).

### Safety of azithromycin 1.5% mass treatment

Questionnaires concerning side effects of treatment were administered by community health workers during daily visits. According to WHO recommendations, if TF is 10% or more in children 1–9 years old, a mass treatment with antibiotic should be conducted throughout the district. Furthermore, as efficacy of the treatment was already assessed in a Phase III study, it was ethical that people of the communities were all treated, so there is no control group to assess the reliability of side effects. The few complaints recorded were local and brief (blurred vision lasting several minutes following instillation of eye drops or transient burning sensation in the eyes). There were no reported serious ocular or systemic side effects.

## Discussion

A statistically significant (p<0.0001) increase in trachoma prevalence was observed between the first prevalence study conducted in 2006 (26%) and the study conducted prior to the first treatment in 2008 (31.5%). It suggests that there was no secular decline trachoma in this area. However, according to the WHO recommendation, as it was unethical to not treat some people of this area, the mass treatment protocol do not planned to have a control group. Thus the prevalence reduction (from 31.5 to 3.1%) of active trachoma among children of this study is likely to be a result of the two mass treatments with azithromycin 1.5% eye drops and prevention campaigns.

By WHO definition, the current prevalence of 3.1% indicates that “trachoma as a blinding disease is being controlled” (TF<5% and TI<0.2%) [4]. The fact that no more TI grade (severity factor of the disease) was observed is particularly encouraging, since TI patients are those most likely to suffer blindness as the disease evolves [14], [15], [2], [12], [16], [17], [18], [19], [20].

Apart from minor complaints, treatment was accepted and well tolerated by both children and adults.

The importance of endemic trachoma in the district of Kolofata justified the mass treatment of the entire population with azithromycin 1.5% eye drops as part of the SAFE strategy and in accordance with WHO recommendations [8]. The apparent coverage decreased between 2008 and 2009 (97% to 89%) is an artefact produced by a change in reporting: in 2009, unlike 2008, only people who had completed all 6 doses were counted.

The most common drug currently used in trachoma mass treatment campaigns is azithromycin 20 mg/kg taken orally. In Niger from 2002 to 2005, SAFE strategy was implemented and three mass treatments using oral azithromycin were performed in 2 districts with 72 villages. Surveys were conducted 3 years apart (before and after program) The prevalence of TF among children decreased from 62.3% and 49.5% to 7.6% and 6.7% in three years [21], a reduction of 89% in one village and 85% in the other. At the same period, in Mali, mass treatment using oral azithromycin was conducted in 7 districts. The prevalence of TI among children decreased from 33% to 2.5% in 3 years [22], a reduction of 92.4%. In a study in Nepal where nearly 40% of children had active trachoma, three rounds of treatment directed at children age 1 to 10 years reduced clinically active trachoma to approximately 13% one year after the first treatment and to 4% one year after the second one. Furthermore, this three annual treatments were successful in reducing infection and disease in these children up to 6 months after the third last treatment (4% of children with active trachoma) [23]. However, is 6 months long enough to determine of the reduction of the disease in a population. To assess this, one study conducted in trachoma-hyperendemic communities in Tanzania determined, after two rounds of mass treatment with oral azithromycin spaced 18 months apart, the rate of trachoma and infection at 5 years, either 3.5 years last treatment. Results showed that 3.5 years after two rounds of mass treatment, trachoma was not eliminated but antibiotherapy appeared to be associate with lower disease prevalence: from 39.2–80.6% (according age groups) at baseline to 7.7–49.1% 5 years after baseline [24].

Considering oral azithromycin studies published, mass treatment with azithromycin 1.5% eye drops, with a reduction about 90% of active trachoma, is at least as effective as treatment with oral azithromycin. Moreover, a phase III clinical trial showed that topical azithromycin 1.5% twice a day for 3 days has a similar efficacy as a single oral 20 mg/kg dose of azithromycin for the treatment of active trachoma in children [25]. The prevalence study planned for 2011, one year after the third annual mass treatment with azithromycin eye drops, should be conclusive.

Finally, trachoma persists where people live in poverty without water, sanitation, [26], [1], [27] and proper waste disposal [26], [27]. Transmission of trachoma occurs where these conditions exist and should be expected to return after antibiotic treatment if the conditions are not changed. Improvements like construction of household pit latrines and hand-dug wells will bring about sustainable elimination of trachoma. However, the Ultimate Intervention Goal for Environmental improvements (UIG-E) defined by the WHO [4] is difficult to set up. Indeed, in 2008 and 2009, only four borehole pumps were installed by the Cameroon government, and two wells were built by OSF. Thus, we are far from UIG-E, i.e. easy access to safe water for 80% of the population [1].

Following encouraging results from the first two mass treatment campaigns with azithromycin 1.5% eye drops, one additional mass treatment campaign was planned. The third campaign took place in January 2010. A fourth study to track the evolution of active trachoma prevalence among children is planned for January 2011.

If the success of these first trachoma mass treatments with eye drops is confirmed, eye drops treatments would be a supplementary tool to fight trachoma in particular in young children and pregnant women.

## Supporting Information

Checklist S1 STROBE Checklist
(0.10 MB DOC)
